# Leptin and Beyond: Actors in Cancer

**DOI:** 10.3390/biom11121836

**Published:** 2021-12-06

**Authors:** Ines Barone, Cinzia Giordano

**Affiliations:** 1 Department of Pharmacy, Health and Nutritional Sciences, University of Calabria, Via P. Bucci, 87036 Arcavacata di Rende, Italy; 2 Centro Sanitario, University of Calabria, Via P. Bucci, 87036 Arcavacata di Rende, Italy

Leptin is a 16-kDa multifunctional, neuroendocrine peptide hormone secreted by adipocytes in proportion to total adipose tissue mass, known to control food intake, energy homeostasis, immune response, and reproductive processes. A growing body of evidence has highlighted that this adipokine, through the binding of its own receptor (ObR) and crosstalk with other pathways (i.e., estrogen, growth factor, and inflammatory cytokine signaling), impacts multiple hallmarks of cancer, including survival, metabolic rewiring, angiogenesis, invasion, and metastasis [1,2,3,4]. Indeed, it has been widely demonstrated that leptin, mainly produced by distant and local adipocytes but also by epithelial tumor cells itself and other cells within the tumor microenvironment (i.e., cancer-associated fibroblasts), is able to affect different aspects of cancer biology in an endocrine, paracrine, and autocrine manner. Accordingly, both clinical and experimental data have indicated the involvement of leptin/ObR axis in the promotion of tumor development and progression, as well as reduction in the efficacy of cancer treatments [1,2,3,4]. Importantly, among adipokines, leptin has been widely recognized as a key driver of the complex network that exists between cancer and obesity, a worldwide recognized health problem [5,6,7,8,9].

As a result, there has been a lot of interest in understanding the molecular mechanisms behind the multiple protumorigenic action of leptin and evaluating whether the signalings triggered by this molecule can function as valuable pharmacological targets to develop more effective anticancer treatments.

The scope of this Special Issue of Biomolecules, “Leptin and Beyond: Actors in Cancer”, was to provide a broad and updated overview of all the aspects that encompass leptin research. The collection includes biochemical and biological studies, bioinformatics analysis, a study of clinical and transcriptomic data of patients, and pharmaceutical approaches emphasizing the role of leptin in the mechanisms of initiation and progression of different type of cancers. Seven contributions were published in this Special Issue, including three original research articles, one communication, and three reviews covering innovative aspects of current research on leptin biology in cancer.

Different experimental approaches, including ‘in vitro’ and ‘in vivo’ models, as well as patient studies focused on the role of leptin in the epithelial–mesenchymal transition (EMT) in various cancers, including breast, prostate, lung, esophageal, and ovarian cancers, have been reviewed by Olea-Flores and colleagues [10]. The review provides a detailed analysis of the latest findings on both the canonical leptin-induced signaling pathways (i.e., PI3K-Akt, JAK-STAT-Akt, and Wnt/β-catenin) and novel and not yet fully described mechanisms that work synergically and converge in the activation or maintenance of EMT. It also discusses evidence of the association of elevated leptin and ObR levels with increased angiogenesis, metastasis, and poor prognosis in obese and overweight cancer patients, highlighting leptin as a key biomolecule that drives EMT and metastatic processes in human cancer.

The involvement of leptin in the mechanisms sustaining more aggressive cancer phenotypes has also been outlined by Panza and colleagues [11]. In the original article, the authors demonstrated the existence of a biologically active interaction between leptin and Notch signaling pathways in the development and progression of Glioblastoma multiforme (GBM). They found that activation of leptin signaling induces growth, motility, and stemness properties in different GBM cell lines through an upregulation of Notch 1 receptor expression and an induction of its downstream signaling effectors. These effects were abrogated by the selective leptin antagonist, the peptide LDFI (Leu-Asp-Phe-Ile) [12] as well as by a specific Notch signaling inhibitor, underlining leptin/Notch crosstalk as a potential novel therapeutic target for GBM treatment.

The study by Gelsomino et al. [13] investigated the contribution of leptin signaling to hormone resistance in breast cancer. The authors identified a role for the obesity-cytokine leptin in sustaining aromatase inhibitor (AI)-resistant growth and progression, using epithelial breast cancer cells surviving long-term treatment with the AI anastrozole (AnaR) as experimental models. Enhanced production of leptin from AnaR cells was discovered as an important mechanism that impacts the crosstalk between breast cancer cells and macrophages within the tumor microenvironment. A quantitative transcriptome profiling of macrophage cells treated with AnaR-derived conditioned media revealed that CXCR4, a well-known receptor regulating cell movement, is involved in AnaR-cell-mediated macrophage chemotaxis. These findings reinforce the role of leptin in human malignancies and suggest the further exploration of the leptin signaling pathway as a predictor of response to endocrine therapies, and as a putative target to be exploited for the development of alternative therapeutic strategies for the treatment of breast cancer patients, especially in the obese setting.

The association of leptin with poor clinical outcomes in clear cells’ renal cell carcinoma (ccRCC) was reported by Fan and colleagues [14]. Clinical information and transcriptomic data of a cohort of 603 ccRCC patients were retrieved from The Cancer Genome Atlas (TCGA) and examined by the authors. Bioinformatic analyses revealed leptin as an independent predictor of a high hazard ratio for ccRCC patients, suggesting its possible prognostic application in clinical management of the disease. In addition, RNA-Seq data from the TCGA allowed the authors to address the molecular mechanism by which leptin promotes cell migration in ccRCC. They found that the planar cell polarity (PCP)/JNK signaling pathway is predictively activated by leptin and ‘in vitro’ studies using a specific pathway inhibitor, confirmed the involvement of this signal in leptin-mediated ccRCC migration ability.

The human small RNA miR-4443 is functionally involved in several tumors and the biology of the immune system, downstream of leptin signaling. The study of Meerson [15], using next-generation sequencing analysis, molecular biology approaches, and knock-out models, indicated that the leptin-responsive miR-4443 was not produced via the canonical Drosha, Exportin 5, and Dicer pathway of microRNA biogenesis in colon cancer cell lines. Indeed, miR-4443 may represent a small regulatory RNA produced by a yet unknown biogenesis pathway, suggesting the need for further studies to dissect this mechanism and understand its potential physiological and pathological functions.

Greco and colleagues [16] provide a comprehensive revision of the modulators of leptin activity, highlighting their potential pharmaceutical applications for the treatment of leptin-related diseases, including cancers. They discuss the latest knowledge of the different classes of leptin-activity modulators: (i) leptin muteins, able to abolish leptin biological activity; (ii) peptide-based leptin receptor antagonist, short-chain peptides consisting of parts of the original leptin sequence; (iii) leptin and leptin-receptor-specific monoclonal antibodies and nanobodies. Indeed, specific anti-leptin-receptor monoclonal antibodies (anti-LR mAbs) have been developed and, due to their high molecular mass, present the advantage of a long circulating half-life along with a good affinity for the receptor. This literature review contributes to a better understanding of the complex biological mechanisms responsible for leptin activities and paves the way for the future development and clinical application of novel and more effective leptin receptor modulators with improved therapeutic action and reduced undesired metabolic side effects.

Despite the large amount of evidence reporting the role of leptin as an important player in many aspects of cancer development and progression, the review by Jiménez-Cortegana and colleagues [17] depicted an additional perspective of leptin activity, underscoring its possible antitumoral role in cancer, which could be part of the so-called “obesity paradox”. They report findings on the dual role of leptin in cancer and clarify its importance as a cornerstone of the immunometabolism in oncology. These latest findings suggest that the pathophysiological processes by which leptin affects cancer biology are not completely clarified, and more research activity is required to decipher the mechanisms by which leptin behaves as a bad or a good actor and for the design of innovative therapeutic tools to modulate leptin signaling, especially in the high-risk obese populations.

To conclude, this Special Issue describes important findings related to the role of leptin in human neoplasia, the underlying molecular mechanisms behind cancer progression and drug resistance, and the potential of different modulators in regulating its activity. All these studies may broaden the knowledge of the function of this critical adipokine and offer new opportunities for the therapeutic management of cancer patients. Surely, research in this field is awaited with interest.
